# SUMO1 modification of methyltransferase-like 3 promotes tumor progression via regulating Snail mRNA homeostasis in hepatocellular carcinoma

**DOI:** 10.7150/thno.42539

**Published:** 2020-04-27

**Authors:** Hongfa Xu, Hao Wang, Wei Zhao, Sirui Fu, Yong Li, Wenjun Ni, Yongjie Xin, Wei Li, Chenzi Yang, Yanyan Bai, Meixiao Zhan, Ligong Lu

**Affiliations:** 1 Zhuhai Interventional Medical Center, Zhuhai Precision Medical Center, Jinan University, Zhuhai People's Hospital, Zhuhai, Guangdong, 519000, China.; 2 Department of urinary surgery, Jinan University, Zhuhai People's Hospital, Zhuhai, Guangdong, 519000, China.; 3 School of Stomatology, Changsha Medical University, Changsha City, Hunan, 410219, China.

**Keywords:** Hepatocellular carcinoma, N6-methyladenosine, SUMOylations, Mettl3, Snail

## Abstract

**Rationale**: Hepatocellular carcinoma (HCC) is one of the leading causes of mortality worldwide. Methyltransferase-like 3 (Mettl3), an RNA N6-methyladenosine (m6A) methyltransferase, has been shown to act as an oncogene in several human cancers. However, the regulatory role of posttranslational modifications of Mettl3 in liver cancer remains elusive.

**Methods**: SUMOylation was analyzed using immunoprecipitation and western blot assays. *In vitro* and *in vivo* biological functions were examined using MTS, colony formation, wound healing, transwell, apoptosis, and viability assays and the BALB/c nude mouse model, respectively. Immunohistochemistry was conducted to evaluate the prognostic value of Mettl3 expression in HCC. The regulatory mechanism of Mettl3 in HCC was investigated by m6A dot blot, immunofluorescence, dual luciferase reporter, protein stability, and RNA stability assays.

**Results**: Mettl3 was found to be SUMOylated by a small ubiquitin-like modifier SUMO1. Further, SUMOylation of Mettl3 was increased upon mitogen stimulation, which correlated with UBC9 upregulation, and was positively correlated with high metastatic potential of liver cancer. Finally, SUMOylation of Mettl3 was found to regulate HCC progression via controlling Snail mRNA homeostasis in an m6A methyltransferase activity-dependent manner.

**Conclusions**: This study revealed a novel mechanism of SUMOylated Mettl3-mediated Snail mRNA homeostasis, identifying the UBC9/SUMOylated Mettl3/Snail axis as a novel mediator of the SUMO pathway involved in HCC progression.

## Introduction

In 2018, hepatocellular carcinoma (HCC) ranked as the sixth most common malignancy worldwide and the fourth leading cause of mortality among patients with malignant tumors 1. The primary risk factors for HCC are predominantly associated with chronic infection with hepatitis B virus (HBV) or hepatitis C virus (HCV), aflatoxin-contaminated foodstuffs, obesity, smoking, type 2 diabetes, among others 1, 2, 3. HCC-associated mortality is generally due to metastasis and postsurgical recurrence 4. Dysregulation of gene transcriptions, including gene mutations, oncogene activation, and inactivation of tumor suppressor genes, are well known to promote tumor metastases. Thus, elucidation of the molecular pathogenesis of HCC is essential to develop new diagnostic and therapeutic inventions and decrease the mortality of this malignancy.

N6-methyladenosine (m6A) is the most abundant internal modification of mRNA in mammals 5. m6A methylation is a dynamic and reversible modification that occurs primarily in 3' untranslated regions (3' UTRs) and near the stop codons 6. Methyltransferase-like 3 (Mettl3), Mettl14, and WTAP comprise the m6A methyltransferases complex 7, 8. m6A methylation is removed by two demethylases ALKBH5 and FTO 9,10. Mettl3 is the critical component of methyltransferases as an S-adenosylmethionine-binding subunit, co-localizes with Mettl4 to form a heterodimer in nuclear speckles, and catalyzes the covalent transfer of a methyl group to adenine (facilitated by other methyltransferase components) 8. Elevated or disturbed Mettl3 expression levels result in changes in the total m6A methylation, which has direct effects on mRNA stability or translation, leading to dysregulated cellular functions. Knockdown of Mettl3 homologs has been shown to lead to defects in gametogenesis 11. Additionally, Mettl3 inactivation leads to a loss of self-renewal capabilities in mouse embryonic stem cells 12. Mettl3 also has been shown to be involved in cancer progression. A decrease in Mettl3 levels results in apoptosis and dysregulation of the p53 signaling pathway in cancer cells 13. High expression of Mettl3 has been directly correlated with metastasis and poor patient prognosis in HCC 14, 15 and disease progression in cervical cancer 16. Consistently, increased Mettl3 expression has been shown to increase invasion of lung adenocarcinoma cells via promotion of oncogenic protein translation 17 and growth inhibition of renal cell carcinoma cells 18. However, it remains to be elucidated whether Mettl3 could epigenetically promote oncogenes in liver cancer through its self-regulatory properties.

SUMOylation, the covalent modification of a protein by a small ubiquitin-like modifier (SUMO) peptide on a lysine residue, has emerged as an important posttranslational modification (PTM) in regulating cellular processes and cancer progression 19. Human genome encodes SUMO1-4 proteins, which are covalently bound to substrate lysine residues through an enzymatic cascade similar to ubiquitination 20, 21. Additionally, the SUMOylation process requires E1-activating and E2-conjugating (UBC9) enzymes 22. UBC9 catalyzes the formation of an isopeptide bond between the C-terminus of SUMO and the amino group of the target lysine. Like other post-translational modifications, SUMOylation is a dynamic and reversible process that regulates target stability, subcellular localization, translation, and protein-protein interaction responsible for a variety of biological functions 20, 21. Increasing numbers of SUMOylated proteins have been shown to be highly expressed in tumor tissues, and activated SUMOylation is closely associated with tumorigenesis 23.

Du et al. reported human Mettl3 modification by small ubiquitin-like modifiers 1 (SUMO1) at K177, K211, K212, K215 both *in vitro* and *in vivo* 24. Notably, SUMOylated Mettl3 levels were significantly lower in the quadruple-lysine mutant than in the single mutants for each lysine, implying that this K-cluster is vital for efficient Mettl3 SUMOylation. SUMOylation of Mettl3 does not affect its stability, subcellular localization, or interaction with METTL14/WTAP, but significant inhibits Mettl3 m6A methyltransferase activity in 293T cells 24. Furthermore, Mettl3 SUMOylation has been preliminary identified to enhance tumor growth in human non-small cell lung carcinoma cell line H1299 24, but the role of Mettl3 SUMOylation in liver cancer progression and the underlying regulatory mechanisms of tumorigenesis have yet to be elucidated. Given the essential role of RNA m6A modification in regulating gene expression and various biological processes, we hypothesize that aberrant m6A modification might also be involved in human carcinogenesis.

Cell polarity normally is necessary to maintain cell integrity and function in tissues; epithelial-to-mesenchymal transition (EMT) is a key process for cancer metastasis. During the EMT process, epithelial cells lose their polarity and acquire invasive capabilities to become mesenchymal stem cells 25-27. Furthermore, EMT is regulated by several transcription factors, including Snail, Slug, Twist, and ZEB1/2 25-27. Snail is a major transcription factor in EMT. The Snail family comprises Snai1, Slug, and Smuc, which all share an evolutionarily conserved role in mesoderm formation in vertebrates 28, and its activity impacts different intracellular signaling pathways, eventually converging on the EMT. This factor confers cancer stem cell-like characteristics in cancer cells and promotes tumor metastasis drug resistance 28. Although Lin et al. reported that Snail is involved in Mettl3-regulated EMT, and m6A in Snail CDS triggers polysome-mediated translation of Snail mRNA in Hela cells 29, there is evidence to suggest that epigenetic modifications regulate tumor progression 23, 30. However, the precise regulatory mechanisms by which posttranslational modifications of Mettl3 impact liver cancer EMT progression remain to be explored.

In this study, we showed Mettl3 SUMOylation by UBC9 with small ubiquitin-like modifier SUMO1 modification. Further, this SUMOylation was increased upon mitogen stimulation and correlated with UBC9 upregulation and induction of EMT-related genes. Mitogen-response SUMOylation of Mettl3 showed a positive correlation to high metastatic potential in liver cancer and regulated EMT progression by controlling Snail mRNA homeostasis via m6A methyltransferase activity. We demonstrated UBC9/SUMOylated Mettl3/Snail axis as a novel mediator of the SUMO pathway involved in liver cancer progression.

## Methods

### Cell lines and Cell culture

Human liver cancer cell lines HepG2, MHCC97H, HEP3B and SMMC-7721 were maintained with 5% CO2 at 37 ºC in high-glucose MEM or DMEM supplemented with 10% heat-inactivated fetal bovine serum (GIBCO USA) supplemented with 10% fetal bovine serum and 100 U penicillin/streptomycin (MDbio, P003-10 g, S007-25 g). For mitogen stimulation, cells were incubated in 5%-20% serum medium for 24 h. All the cells were purchased from ATCC and the culture period did not exceed two months.

### Quantitative real-time PCR

Total RNA of liver cancer cells were extracted using Trizol reagent (Invitrogen, 15596018, CA, USA), and were reverse-transcribed using a RevertAid First Strand cDNA Synthesis Kit (Thermo, K1622). The cDNA was used as the template for the subsequent qRT-PCR analysis using PrimeScriptTMRT reagent Kit with gDNA Eraser (Code No. RR047A). GAPDH was used as the normalization gene. The mRNA levels of the genes were reckoned as two power values of ΔCt (the Ct of the GAPDH minus the Ct of the target gene). Primers of targeted genes were as follow: GAPDH, forward 5'-GTCTCCTCTGACTTCAACAGCG-3' and reverse 5'-ACCACCCTGTTGCTGTAGCCAA-3', CDH1, forward 5'-GCCTCCTGAAAAGAGAGTGGAAG-3' and reverse 5'-TGGCAGTGTCTCTCCAAATCCG-3', Snail, forward 5'-TGCCCTCAAGATGCACATCCGA-3' and reverse 5'-GGGACAGGAGAAGGGCTTCTC-3', UBC9, forward 5'-ATCCAAGACCCAGCTCAAGCA G-3' and reverse 5'-TTGACGATGCCACAAGGTCGCT-3', Mettl3, forward 5'-CTATCTCCTGGCACTCGCAAGA-3' reverse 5'- GCTTGAACCGTGCAAC CACATC-3'.

### Lentivirus and Plasmids

Plasmid construction was performed according to previously described standard method 31. In brief, human full-length and mutatuions Mettl3 cDNAs were subcloned and linked into pEZ-Lv203. His-SUMO-1/2/3 and HA-UBC9 and Snail plasmids were purchased from GeneCopoeia,Inc. Infection of lentivirus were performed according to previously described standard method 32. Plasmids were transfected by LipofectamineTM 2000 Transfection Reagent (InvitrogenTM, 11668019).

### Small interfering RNA transfection

siRNA target of Mettl3, UBC9 and scramble control were purchased from RIBOBIO. Transfection of siRNA duplexes was transfected using LipofectamineTM 2000 Transfection Reagent. The targets of the UBC9 siRNAs were 5'-TTGGCAGTAAATCGTGTAGGCC-3'(si#1) and 5'-ATTTAGAAGTTCCTGTATTCCT-3'(si#2), siMettl3 sequencing were 5'-GACTGCTCTTTCCTTAATA-3'(si#1) and 5'-GGACTCGACTACAGTAGCT-3'(si#2).

### Cell proliferation assays and colony-formation assays

Colorimetric MTS assays (Promega; G3582) were performed to determine the growth and viability of the liver cancer cells, as previously reported 32. Briefly, 800 cells/well were treated in a 96-well culture plate (Corning) in triplicate. Then harvested the parallel culture plates, and added 20 μL MTS solution (promega, G3580) to each well. The optical density value was determined at 490 nm after incubating the sulution for 2 h.

For the colony formation experiments, 500-800 cells/well were bred in a 6-well plate (Corning). After 10 days, the resulting colonies were fixed in paraformaldehyde for 10 min and stained with 1% crystal violet. Only the colonies that included more than 50 cells were considered.

### Analysis of cell motility

Cell motility was detected by using the transwell and wound healing assays. Migration assays were conducted using chambers without Matrigel (Falcon, 353,097) based on the manufacturer's protocol. Briefly, cells in 200 uL of serum-deprived medium were seeded to the upper chamber and allowed to migrate to the other side of the membrane. After 24 h, the chambers were fixed in paraformaldehyde for 10 min and stained with crystal violet. Images were captured from each membrane and using the ImageJ software count for the metastatic cell numbers. The wound healing assay was performed using a 200 uL pipette tip to scratch the cell layer, and the wound healing rate was recorded after 0, 36 and 48 h. All experiments were independently repeated three times.

### Cell apoptosis and viability assay

For cell viability and apoptosis assays, 48 h after transfection, cells were harvested and seeded with requested concentration, and then viability and apoptosis were assessed using ApoLive-Glo Multiplex Assay Kit (Promega) following the corresponding manufacturer's manuals.

### Immunoprecipitation assay and western blot analysis

After cells were stimulated for the indicated time periods, they were washed with PBS and lysed in cold RIPA buffer (Thermo Scientific, 89900) containing a protease and phosphatase inhibitor mixture (Beyotime Biotechnology) for 20 min at 4 °C and centrifuged at 13,000 rpm for 30 min. Then protein concentration was assessed with the BCA protein assay kit (Pierce Biotechnology) following the manufacturer's instructions. Subjecting equal amounts of protein mixed with sample loading buffer (5X; Beyotime) were separated by SDS-PAGE using Bis-tris polyacrylamide gels and transferred to polyvinylidene difluoride membranes (Millipore). After blocking with 5% skim milk in tris-buffered saline tween-20, the membranes were incubated with the primary antibodies overnight at 4 °C and then with the horseradish peroxidase-conjugated secondary antibodies at room temperature for 1 h. For immunoprecipitation, samples were lysed in lysis buffer (25 mM Tris-HCl pH 7.4, 150 mM NaCl, 1 mM EDTA, 20 mM N-ethylmaleimide, 1% NP-40). Aliquots (1000 ug) of cell lysates were incubated with primary antibody overnight; the next day, samples were incubated with Protein A/G PLUS-Agarose (santa cruz, Sigma) at 4 °C for 2 h. Samples were washed with immunoprecipitation lysis buffer three times and the proteins were extracted from the Agarose in SDS sample buffer containing reducing agent and heating at 95 °C. Then the eluted protein samples were separated on SDS-PAGE. The primary antibodies, including anti-GAPDH, anti-LaminB1, anti-His, anti-HA, anti-Flag, anti-MMP2 (from proteintech), anti-Mettl3 (from abcam, Bethyl); anti-m6a (from Synaptic Systems); SUMO-1 and SUMO2/3 (from abcam); anti-MMP9, anti-E-cadherin and normal rabbit IgG (from CST); UBC9, Snail and normal mouse IgG (from santa cruz); anti-rabbit, Anti-mouse peroxidase-conjugated secondary antibodies (from proteintech).

### m6A dot blot assay

mRNA was enriched using Dynabeads mRNA DIRECT Purification Kit (InvitrogenTM, 61012). After denaturation at 65°C for 5 min and chilling directly on ice, 400 ng, 200 ng, and 100 ng of samples mixed with the ice-cold 20*SSC buffer (Sigma-Aldrich) were spotted to an Amersham Hybond-N+ membrane (GE Healthcare). Following, the membrane was dried for 10 min, UV crosslinked for 10 min, and washed with PBST. Then, it was stained with 0.02% Methylene blue (Beyotime Biotechnology) and scanned to indicate the total amount of input mRNA. After blocking with 5% BSA for 1 h, the membrane was incubated with m6A antibody (Synaptic Systems) overnight at 4 °C. Finally, the membrane was incubated with the HRP-conjugated anti-rabbit immunoglobin IgG secondary antibody for 1 h before visualization by an imaging system (Promega).

### Immunofluorescence and immunohistochemistry

Cells were transfected with the respective plasmids and cultured on confocal dishes, fixed with 4% paraformaldehyde for 20 min, and permeabilized with 0.1% Triton X-100 for 20 min. Cells were blocked in 5% BSA and incubated with primary antibody at 4 °C overnight followed by incubation with secondary antibodies. Then cells were mounted in medium with 4',6-diamidino-2-phenylindole (DAPI) to visualize nuclei. Images were obtained with a confocal microscope with an oil immersion 63X lens (Olympus).

Paraffin sections were deparaffinized for 2 h at 60 °C, then rehydrated through an alcohol series followed by sodium citrate buffer. The sections were treated in 3% hydrogen peroxide and subsequently blocked with 5% normal goat/mouse serum. Finally, the sections were incubated with appropriate primary antibodies at 4 °C overnight. Immunohistochemistry staining was performed with horseradish peroxidase (HRP) conjugates using Diaminobenzidine (DAB) detection. Images were taken with an Olympus microscope.

### Nuclear and cytoplasmic protein extraction

The nuclear and cytoplasmic proteins components of HCC cells were isolated using NE-PER nuclear and cytoplasmic extraction reagents (Thermo Scientific), according to the manufacturer's protocol. GAPDH or LaminB1 served as controls for the cytosolic or nuclear fractions, respectively.

### Animal experiments

All animal experiments were approved by the Institutional Animal Care and Usage Committee of Zhuhai People's Hospital. Four-week-old female BALB/c nude mice were obtained from the Guangdong experimental animal center (Guandong, China). For the tumorigenesis assay, BALB/c nude mice were randomly divided into three groups (n = 5 for each group). Following, 1×10^6^/mouse cells were suspended in 50 μL of serum-free DMEM and subcutaneously injected into the flank of each mouse. Tumor diameters and volume (length × width^2^ × 0.5236) were calculated every other day using calipers. For lung metastasis experiment, 3×10^5^ cells suspended in 100 μL serum-free DMEM were intravenously injected through the tail vein of the mice. Lung metastatic nodules were calculated after six weeks when the mice died of cachexia. Lungs were excised for hematoxylin and eosin staining.

### RNA decay assay

To evaluate the RNA stability in Mettl3-wild-type and Mettl3-mut-type HCC cells, Actinomycin D (AbMole, M4881) was added to cells with a final concentration of 5 μ g/mL. Then cells were collected after 0, 30, 60, 120, 240 min respectively, and RNA was isolated for RT-PCR to calculate the relative abundance of Snail mRNA.

### Protein stability

To measure target protein stability, HCC cells were feeded with cycloheximide at a final concentration of 100 μg/ml (AbMole, M4879) during indicated times. Then the expression level of Snail was determined by western blot.

### Luciferase reporter assay

Snail promoter sequences were cloned into pEZX-PL01 control vectors comprising firefly luciferase (F-luc) and renilla luciferase (R-luc). Luciferase assay was performed using Luc-Pair™ Duo-Luciferase HS Assay Kit (GeneCopoeia), according to the manufacturer's instructions. Briefly, pre-treated HCC cells were co-transfected with Mettl3-wild-type or Mettl3-Mut-type and pEZX-PL01 reporter plasmids in a 12-well plate. After transfection for 6 h, cells were re-seeded into a 96-well plate. After a 36-h incubation, cells were harvested for analysis using the Dual-Glo Luciferase Assay system. F-luc activity was normalized to R-luc activity to evaluate the luciferase or transcriptional activity. Each group was assessed in triplicate.

### Statistical analysis

All data are represented as mean ± SD. Statistical analyses were performed using SPSS (version 17) and GraphPad Prism version 7.0 (USA, GraphPad Software) software. Chi-squared tests were used to analyze the relationship between Mettl3 expression and clinicopathological status. Overall survival based on Mettl3 and Snail expression were analyzed using the Kaplan-Meier survival and log-rank test. Data were analyzed under the Student's t-test (2-tailed) or ANOVA methods. *P*-value of less than 0.05 was considered as statistically significant.

## Results

### Mitogen induces Mettl3 conjugated to SUMO-1

**Figure 1:**
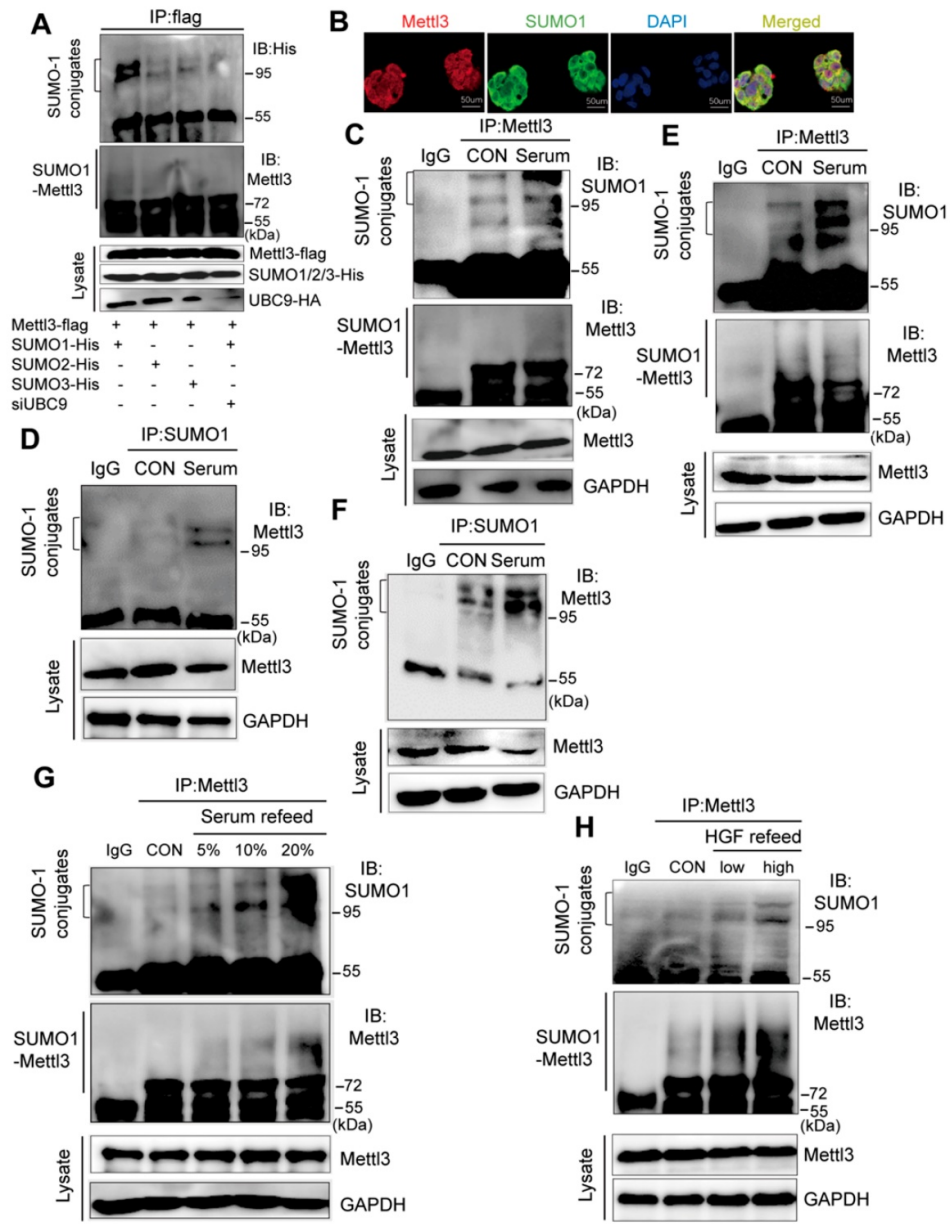
** Mitogen stimulates Mettl3 conjugated to SUMO-1. (A)** Mettl3 was overexpressed in MHCC97H cells by transfection with Flag-tagged wild type Mettl3. Subsequently, the cells were transfected with His-SUMO1, -SUMO2, -SUMO3, and HA-Ubc9 or siUBC9. Immunoprecipitation (IP) and western blotting analyses were performed with the indicated antibodies for the SUMOylation assay. **(B)** Confocal immunofluorescence of endogenous Mettl3 and SUMO1 proteins in MHCC97H cells (Mettl3, green; SUMO1, red). Both Mettl3 and SUMO1 were observed in the nucleus and cytoplasm. Scale bars, 50 μm.** (C-F)** Representative IP immunoblot analysis was conducted with the anti-Mettl3 or anti-SUMO1 antibody and whole-cell extracts from HepG2 or MHCC97H cells incubated in serum-containing or serum-free medium for 24 h. **(C** and **E)** IP analysis was performed with anti-Mettl3 antibodies in HepG2 and MHCC97H cells. **(D** and **F)** IP analysis was performed with anti-SUMO1 antibodies in HepG2 and MHCC97H cells. **(G)** IP immunoblotting analysis examining SUMOylation of endogenous Mettl3 from whole-cell extracts after the addition of 5%, 10%, and 20% serum with anti-Mettl3 antibody or normal IgG, followed by western blotting with the indicated antibodies. **(H)** IP immunoblotting analysis examining SUMOylation of endogenous Mettl3 from whole-cell extracts after the addition of low (10 ng/ml) or high (20 ng/ml) concentration HGF with anti-Mettl3 antibody or normal IgG, followed by western blotting with the indicated antibodies.

SUMOylation consensus motif primarily consists of ψKXE, where ψ is a large hydrophobic residue, K is the acceptor lysine residue, X represents any residue, and E is an acidic residue, which is not inclusive of all discovered SUMO1 conjugation sites 19, 22. To explore the interaction between SUMO and Mettl3, we performed co-immunoprecipitation assays and immunofluorescent staining. We transiently transfected Mettl3-Flag and the SUMO-conjugating enzyme E2 Ubc9 siRNA together with His-tagged SUMO1, SUMO2, or SUMO3 into liver cells. The result showed that precipitated protein complexes using a Flag-tagged antibody included SUMOylated Mettl3, which was primarily modified by SUMO1 and very weakly modified by SUMO2 and SUMO3 (Figure. 1A). Moreover, the precipitated protein complexes included more SUMOylated Mettl3-Flag in liver cancer cells expressing Mettl3-Flag, SUMO1, and scramble siRNA than in cells expressing Mettl3-Flag, SUMO1, and Ubc9 siRNA, indicating that UBC9 is required for Mettl3 SUMOylation (Figure 1A). Additionally, in our immunofluorescent experiments, an overlapping signal (yellow) of Mettl3 (red) and SUMO1 (green) was observed in the cytoplasm and nucleus of liver cancer cells (Figure 1B), indicating that Mettl3 can be conjugated to SUMO-1.

To investigate the possible regulatory role of Mettl3 SUMOylation in liver cancer, we examined mitogen-response SUMOylation of Mettl3 in MHCC97H cells. First, these cells were lysed in denatured lysis buffer. Subsequently, the protein complexes were precipitated with a control IgG or Mettl3, followed by immunoblotting of anti-SUMO1 and anti-Mettl3. When serum-deprived quiescent cells were stimulated with serum, Mettl3 SUMOylation was significantly increased compared to the control group (Figure 1C). Additionally, IP assay with anti-SUMO1 and control IgG followed by immunoblotting of anti-Mettl3 showed an increased Mettl3 SUMOylation upon serum stimulation (Figure 1D). Consistently, similar results were observed using HepG2 cells (Figure 1E-F). In addition, liver cancer cells were treated with varying concentrations of fetal bovine serum. Immunoblotting analysis indicated that exposure to serum for 24 h resulted in significantly increased Mettl3 SUMOylation in a serum dose-dependent manner (Figure 1G). Moreover, hepatocyte growth factor (HGF), a known mitogenic signal associated with invasive program and metastasis during tumor progression in HCC, was included as another mitogen to stimulate cells, and the mitogen-response SUMO-conjugated Mettl3 was examined by immunoprecipitation with anti-Mettl3 specific antibody. Endogenous Mettl3-SUMO-1 was more significant in the cells following high concentration of HGF treatment (Figure 1H). We also investigated whether Senps, an SUMO1 modification-specific protease, can regulate SUMO1 modification of Mettl3 under mitogenic response. After serum induction, the SUMO1 antibody-precipitated protein complexes containing endogenous SUMOylated Mettl3 bands were significantly increased, which were almost completely eliminated by treatment with Senps (Figure S1). These results suggest that mitogen stimulation was able to trigger Mettl3 SUMOylation and enhance SUMO1 conjugation to Mettl3 in liver cancer.

### SUMOylation of Mettl3 correlates with UBC9 upregulation in response to mitogen in liver cancer cells

**Figure 2:**
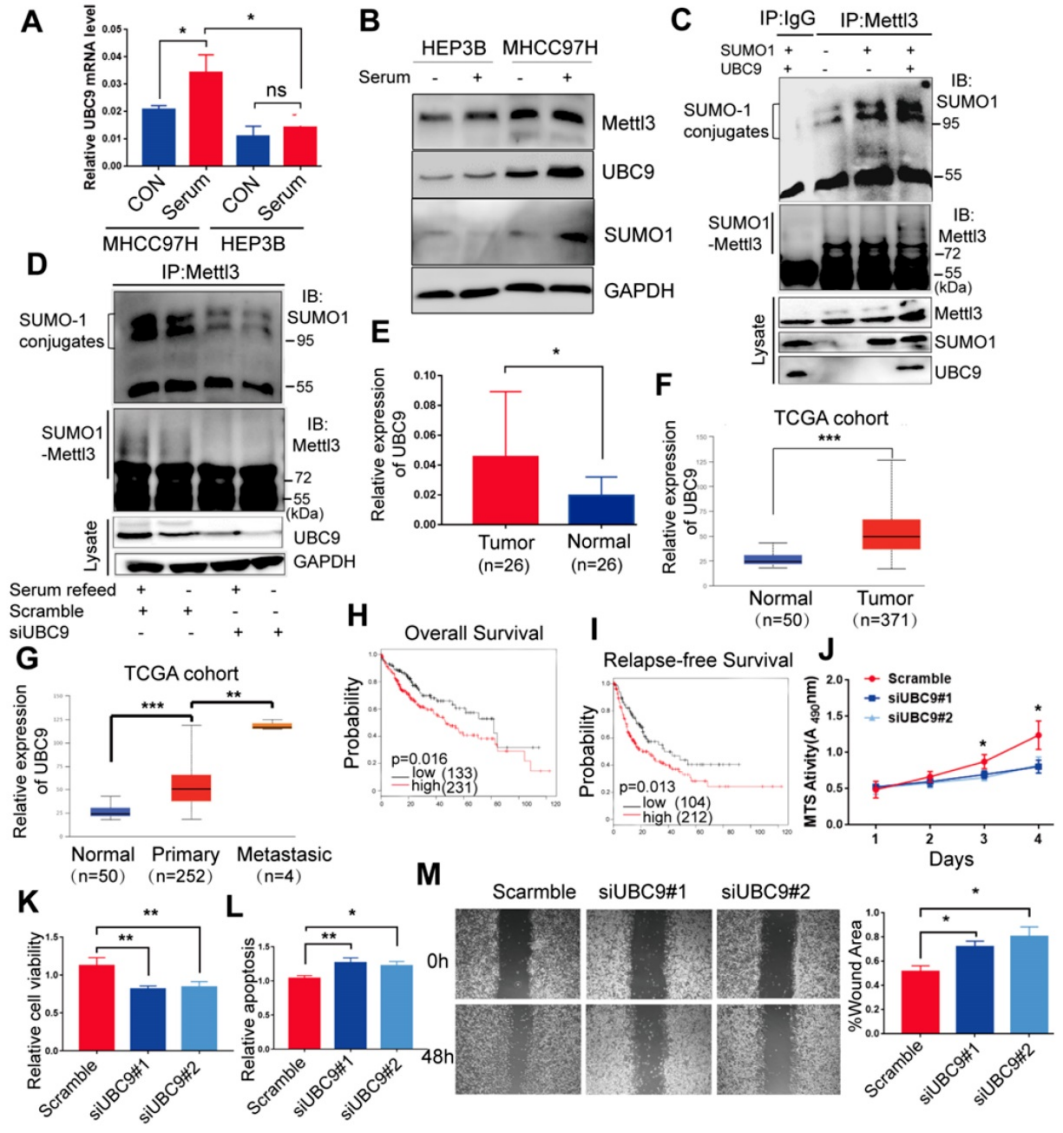
** Mettl3 SUMOylation correlates with UBC9 upregulation in response to mitogen in liver cancer cells. (A)** qRT-PCR analysis of HEP3B or MHCC97H cells fed in the presence of serum or serum-starved for UBC9 expression. **(B)** Immunoblot analysis of whole-cell lysates (WCL) from HEP3B or MHCC97H cells stimulated in serum-containing or serum-free medium for Mettl3, UBC9, and SUMO1 expression. **(C)** MHCC97H cells were transfected with SUMO1 in combination with Ubc9, followed by IP and western blotting with the indicated antibodies. **(D)** IP immunoblot analysis was conducted with an anti-Mettl3 antibody and whole-cell extracts from MHCC97H cells stimulated with or without serum in the presence of scramble or Ubc9 siRNA. Twenty-four hours after transfection, cells were harvested and subjected to co-immunoprecipitation with the anti-Mettl3 antibody, followed by western blotting with the indicated antibodies. **(E)** UBC9 upregulation in HCC and its matched adjacent normal tissues of 26 patients was analyzed (mean ± s.d., * P < 0.05). **(F** and **G)** UBC9 expression level was elevated in 371 HCC tissues compared with 50 normal liver tissue samples** (F)** and positively correlated with metastatic HCC tissues **(G)** in the TCGA profile, based on the ualcan database (http://ualcan.path.uab.edu/index.html). **(H** and **I)** Overall survival analysis **(H)** and relapse-free survival **(I)** based on UBC9 expression in HCC, according to kmplot online database (http://kmplot.com/analysis/). **(J-M)** Effects of decreased UBC9 expression on cell proliferation **(J)**, viability **(K)**, apoptosis **(L)**, and migration **(M)** in MHCC97H cells. Data are presented as mean ± s.d. * p < 0.05, ** p < 0.01, ***<0.001; Student's t-test.

As UBC9 serves as the single E2-conjugating enzyme, it is essential for SUMO conjugation to its substrates. Therefore, we hypothesized that upregulation of Mettl3 SUMOylation in response to mitogen stimulation in liver cancer might be due to UBC9 upregulation. To verify this, we examined the mRNA and protein levels of UBC9 in MHCC97H and HEP3B cells. Compared to HEP3B cells, levels of UBC9 basal mRNA and protein, and SUMO1 protein, were significantly higher in MHCC97H cells, which were exacerbated upon mitogen stimulation (Figure 2A-B). Consistently, Mettl3 SUMOylation and UBC9 protein level in serum-deprived quiescent cells were dramatically elevated upon serum stimulation (Figure S2). However, there were no differences in Mettl3 expression in HCC97H or HEP3B cells following mitogen stimulation (Figure 2B).

Furthermore, we evaluated the role of UBC9 in the mitogen-response Mettl3 SUMOylation. We transfected SUMO1 with or without Ubc9 plasmid to verify whether endogenous Mettl3 could be SUMOylated by SUMO1. As expected, endogenous Mettl3 was modified by SUMO1, which was significantly enhanced by overexpression of UBC9 (Figure 2C). By contrast, depletion of UBC9 impeded SUMO-1 conjugation on Mettl3 in liver cancer cells upon mitogen stimulation, suggesting that UBC9 is both necessary and sufficient for mitogen-induced Mettl3 SUMOylation (Figure 2D). The knockdown efficiencies of UBC9 siRNAs were verified (Figure 2D).

We further investigated the clinical roles of UBC9 in 26 human non-cancerous and 26 HCC tissue samples. RT-qPCR analysis showed that the mRNA level of UBC9 in liver cancer tissues was significantly higher than that in the non-cancerous tissues (Figure 2E). We also checked the Cancer Genome Atlas (TCGA) database, and found that UBC9 was upregulated in HCC (Figure 2F). Furthermore, UBC9 expression is positively correlated with the metastatic potential of HCC (Figure 2G), and inversely correlated with overall survival or relapse-free survival (Figure 2H-I). Further, *in vitro* function studies were performed to investigate the role of UBC9 in liver cancer. As shown in Figure. 2J-M, silencing of UBC9 with siRNA decreased cell growth/proliferation, viability, and EMT, while promoting apoptosis in liver cancer cells.

### Mettl3-SUMO1 conjugates are essential for its oncogenic properties and correlates with Snail upregulation in liver cancer cells

**Figure 3:**
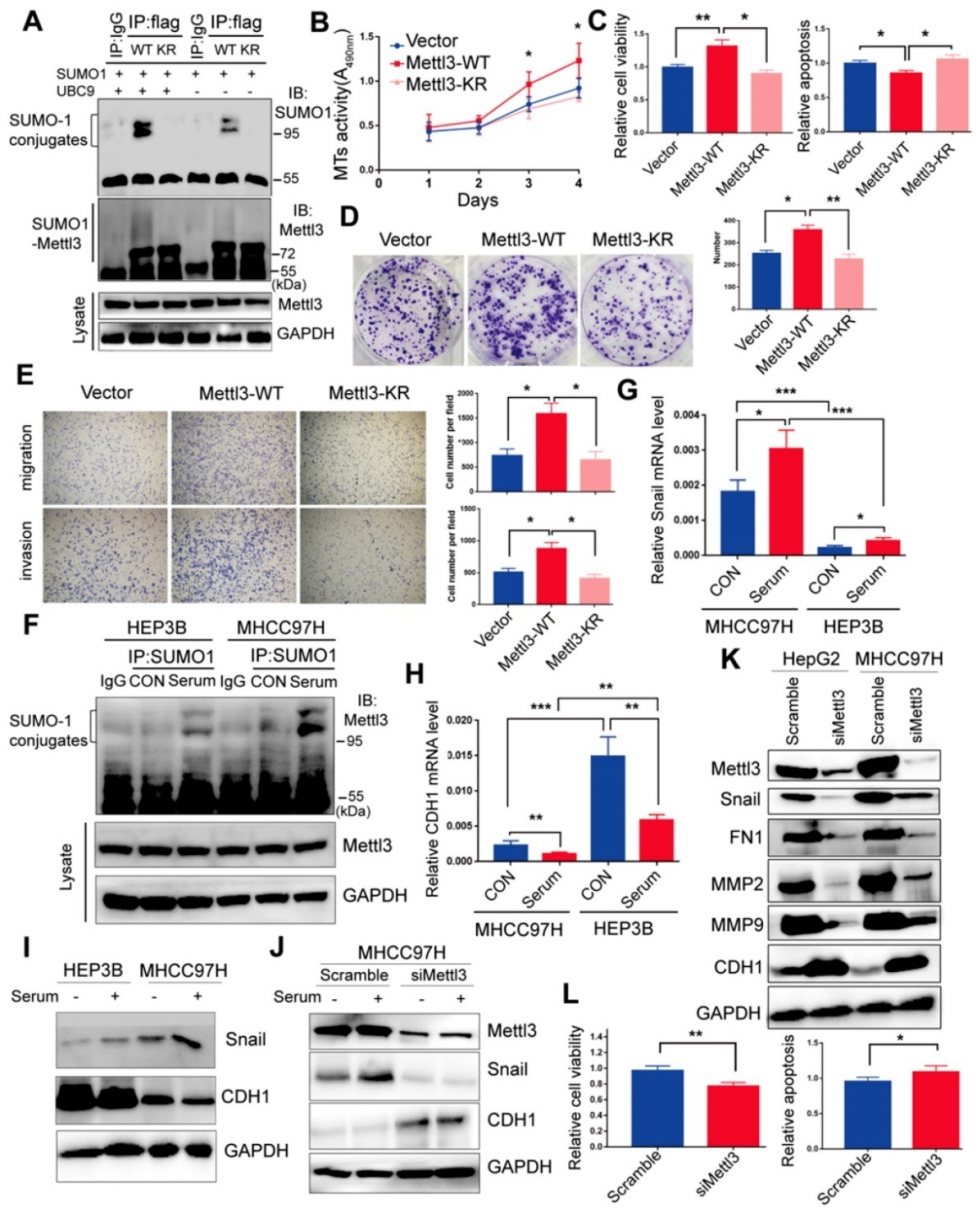
** Mettl3-SUMO1 conjugates are essential for its oncogenic properties and correlates with Snail upregulation and is positively associated with high metastatic potential of liver cancer cells. (A)** IP immunoblot analysis was conducted with an anti-Flag antibody and whole-cell extracts from Flag-tagged wild type Mettl3, or SUMOylation-defective Mettl3-^K177/K211/K212/K215^-Flag (KR)-expressing MHCC97H cells transfected with or without His-SUMO1 and HA-UBC9. **(B-E)** Effects of vector-, Mettl3-WT-, or Mettl3-KR-mutant-expressing cells on cell proliferation **(B)**, viability and apoptosis **(C)**, colony formation **(D)**, migration and invasion **(E)** in MHCC97H cells. **(F)** IP immunoblot analysis was conducted with the anti-SUMO1 antibody and whole-cell extracts from HEP3B or MHCC97H cells stimulated with or without serum. Cells were harvested and subjected to co-immunoprecipitation with the anti-SUMO1 or control IgG antibody, followed by western blotting with the indicated antibodies. **(G** and **H)** qRT-PCR analysis of HEP3B or MHCC97H cells stimulated with serum or deprived of serum for Snail **(G)** and CDH1 **(H)** expression. **(I)** Immunoblot analysis of WCL from HEP3B or MHCC97H cells stimulated with serum or deprived of serum for Snail and CDH1 expression. **(J)** Immunoblot analysis of WCL from scramble or siMettl3-expressing cells stimulated with serum or deprived of serum for Mettl3, Snail and CDH1 expression. **(K)** Knock-down of endogenous Mettl3 impaired expression of EMT-related genes. Immunoblot analysis of WCL from scramble or siMettl3-expressing cells. **(L)** Effects of scramble or siMettl3-expressing cells on cell viability and apoptosis. Data are presented as mean ± s.d. * p < 0.05, ** p < 0.01, ***<0.001; Student's t-test.

Du et al. reported K^177/211/212/215^ as major SUMOylation acceptor sites of Mettl3 24. To evaluate Mettl3 kinase activity in SUMOylation site mutants, we generated a quadruple-lysine mutant K^ 177/211/212/215^ R (KR) of the Mettl3 protein, in which the lysine residues (Ks) were mutated to arginine (R). We found that the mutant Mettl3-KR had much less SUMOylation than Mettl3-WT, which was further reduced following UBC9 overexpression (Figure 3A). Multiple studies have suggested that Mettl3 functions as a potential tumor promoter, and SUMOylation can regulate activities of some SUMO-targeted enzymes and target proteins 20, 21. Therefore, we performed *in vitro* functional studies to investigate the role of Mettl3 SUMOylation in liver cancer. As shown in Figure 3B-E, transient transfection with Mettl3-WT, but not Mettl3-KR, promoted cell growth/proliferation, epithelial-mesenchymal transition (EMT), and viability, while decreasing apoptosis in liver cancer, indicating that Mettl3 SUMOylation is essential for its oncogenic properties.

Moreover, our study indicated that mitogen stimulation enhanced SUMO1 conjugation of Mettl3, thus promoting liver cancer progression. Thus, to explore the regulatory role of mitogen-responsive SUMOylation of Mettl3 and its relation to liver cancer metastatic potential, MHCC97H and HEP3B cells with high and low metastatic potential 33, 34, respectively, were compared. After serum-deprived quiescent cells were induced with serum, the SUMO1 antibody-precipitated protein complexes containing endogenous SUMOylated Mettl3 were significantly increased in MHCC97H cells compared to HEP3B cells (Figure 3F). Similar results were also observed in HCCLM3 cells compared to PLC/PRF/5 cells (Figure S3).

Furthermore, Snail mRNA expression was upregulated, but CDH1 was downregulated, in MHCC97H cells with or without serum stimulation (Figure 3G-H). Correspondingly, Snail protein expression levels was upregulated, but CDH1 was downregulated in MHCC97H cells (Figure 3I), which was further exacerbated following mitogen stimulation. However, knockdown of Mettl3 expression abolished serum-stimulated expression of Snail protein (Figure 3J). These results also demonstrated that MHCC97H cells had higher metastatic ability than HEP3B cells.

Moreover, after Mettl3 knockdown in liver cancer cells, Western blot analysis validated that Mettl3 knockdown decreased the protein level of Snail, MMP2, MMP9, and FN1, but increased that of E-Cad, in both HepG2 and MHCC97H cells (Figure 3K). Additionally, *in vitro* functional assays showed that siMettl3-mediated knockdown of Mettl3 decreased cell viability, while promoting apoptosis in liver cancer cells (Figure 3L), suggesting that Mettl3 was involved in the progression of HCC. Collectively, these results showed that the mitogen-response SUMOylation of Mettl3 was positively correlated with high metastatic potential liver cancer, which was regulated by Snail.

### SUMOylation-deficient Mettl3 inhibits Snail accumulation and regulates liver cancer progression *in vivo* and *in vitro*

**Figure 4:**
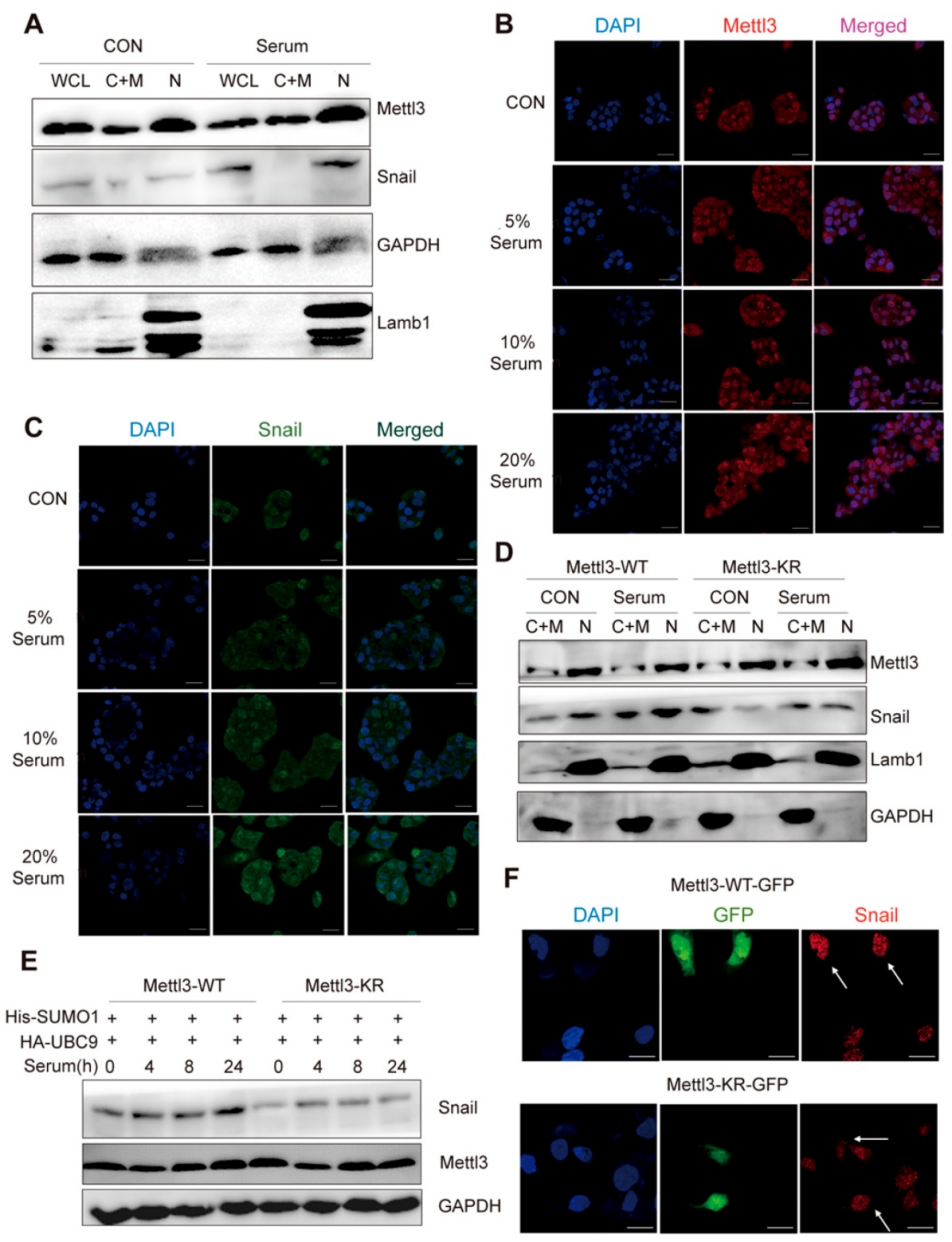

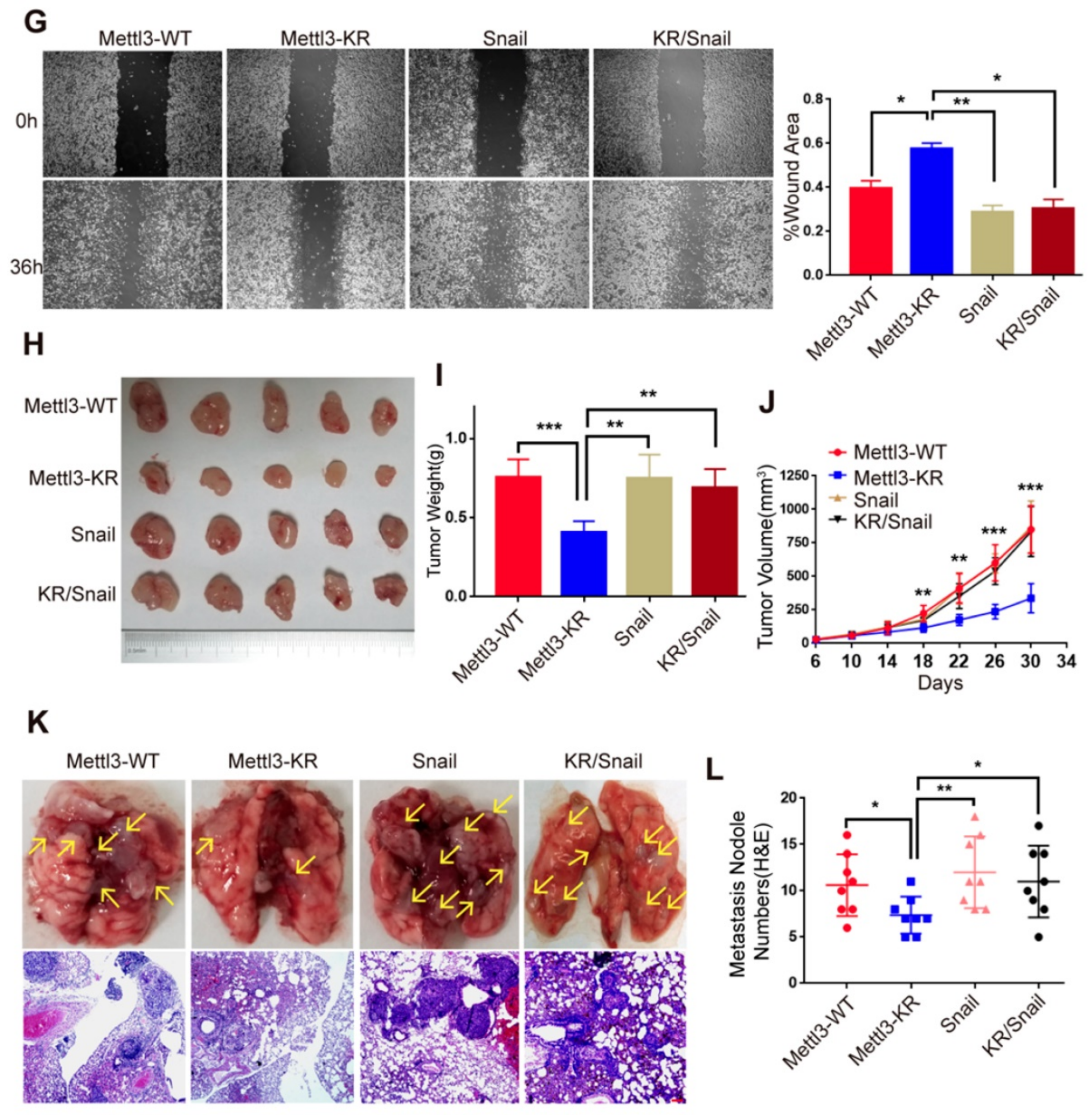
** SUMOylation-deficient Mut-Mettl3-KR disrupts Snail recruitment and regulates liver cancer progression *in vivo* and *in vitro*. (A)** Whole-cell lysates (WCL), cytosolic/membrane (C+M), and nuclear (N) fractions from MHCC97H cells stimulated with serum or serum-free medium were subjected to western blotting to examine the endogenous Mettl3 and Snail expression. LMNB1 and GAPDH were used as loading control of C+M and N, respectively. **(B** and **C)** Endogenous expression level and localization of Mettl3 **(B)** and Snail **(C)** in HepG2 after the addition of 5%, 10%, and 20% serum were confirmed by confocal immunofluorescence. Scale bars, 50 μm. **(D)** Immunoblot analysis of C+M and N fractions from Mettl3-WT or Mettl3-KR-expressing MHCC97H cells incubated in serum or serum-free medium Mettl3 and Snail protein levels were confirmed by western blotting. LMNB1 and GAPDH were used as loading control of C+M and N, respectively. **(E)** Immunoblot analysis of WCL from Mettl3-WT or Mettl3-KR-expressing MHCC97H cells transfected with different plasmids and treated with serum for different time periods. **(F)** MHCC97H cells were transfected with GFP-tagged WT or KR (green) and immunostained with Snail antibodies, followed by incubation with Alexa Fluor 546-tagged (red) secondary antibody for visualization. Further, the nuclei were counterstained with DAPI. Scale bars, 25 μm. **(G)** Effects of Mettl3-WT-, Mettl3-KR-, Snail and Mettl3-KR/Snail-expressing cells on cell migratory ability were detected by wound scratch assay. The area of wound scratch in response to serum stimulation is shown, with 100% representing the control at 0 h. **(H)** Subcutaneous injection of Mettl3-WT-, Mettl3-KR-, Snail and Mettl3-KR/Snail-expressing cells into nude mice. **(I** and **J)** Mouse weights **(I)** and tumor volumes **(J)** were measured every four days. **(K)** Mettl3-WT-, Mettl3-KR-, Snail and Mettl3-KR/Snail-expressing cells were injected into nude mice by tail vein injection. Representative images of metastatic lung tumors (upper panel) and the H&E staining results are shown (lower panel). Scale bars, 100 μm. **(L)** The numbers of microscopic metastatic nodules in the lung sections were quantified. Data are presented as mean ± s.d. * p < 0.05, ** p < 0.01, ***<0.001; Student's t-test.

As SUMOylation is a key post-translational modification for maintaining proper Mettl3 localization and normal function, and SUMOylation can regulate the activity of some SUMO-targeted proteins 20, 21, the possible regulatory role of Mettl3 SUMOylation to modulate Snail expression in response to mitogen was further evaluated. We found that mitogen stimulation did not influence the localization of endogenous Mettl3, but significantly elevated Snail protein levels compared to the serum-deprived group (Figure 4A). Further, in the immunofluorescent experiments, mitogen stimulation increased Snail expression in a dose-dependent manner, but not Mettl3 (Figure 4B-C). In Mettl3-WT-expressing cells, Snail level in the nuclear fraction was increased following serum stimulation. However, this increase was not observed in Mettl3-KR mutant-expressing cells, and no changes in Mettl3 were found between the Mettl3-WT-expressing and Mettl3-KR mutant-expressing groups with serum or deprived of serum stimulation (Figure 4D).

However, serum treatment of cells at different times stimulated SUMO1 conjugation of Mettl3 with exogenous SUMO1 and Ubc9 in Mettl3-WT but not in Mettl3-KR-expressing cells (Figure 4E), similar to the effect found in Figure. 4D. Consistently, in transiently transfected liver cancer cells with Mettl3-WT-GFP or Mettl3-KR-GFP plasmid (green), following by immunostaining with Snail antibodies and Alexa Fluor 546-tagged (red) secondary antibody for visualization, SUMOylation-deficient Mettl3 was found to disrupt Snail accumulation in liver cancer compared to the Mettl3-WT-expressing group (Figure 4F).

Next, we examined the effect of Mettl3 SUMOylation regulation of Snail- mediated liver cancer progression *in vivo* and *in vitro*. Since SUMOylation-deficient Mettl3 limited Snail accumulation, Snail expression was increased in the Mettl3-KR mutant-expressing group, which showed that Snail overexpression antagonized the suppression of wound healing through Mettl3 SUMOylation deficiency in liver cancer cells (Figure 4G).

On the contrary, Snail knockdown attenuated the facilitating effect of Mettl3 on wound healing via METTL3 overexpression in HCC cells (Figure S4), suggesting that the effect of Mettl3 on the progression of liver cancer cells may depend on Snail expression. We then performed an animal xenograft model and tail vein injection to analyze subcutaneous nodules and lung colonization. Mettl3-WT-, Mettl3-KR-, Snail- and Mettl3-KR/Snail-expressing cells were injected into nude mice subcutaneously or intravenously through the tail vein. As shown in Figure 4H-J, tumor growth and weight were reduced in the Mettl3-KR cells group. Once elevated, Snail expression attenuated the suppression effect of SUMOylation-deficient Mettl3. As for the lung colonization, the number of lung tumors derived from Mettl3-KR cells was decreased compared to another three groups. However, Snail overexpression reduced the suppression effect of Mettl3-KR cells on *in vivo* lung colonization (Figure 4K-L). Therefore, these data suggest that Mettl3 SUMOylation was involved in liver cancer progression *in vivo* and *in vitro* by affecting Snail accumulation.

### SUMOylation of Mettl3 regulates Snail mRNA homeostasis via m6A methyltransferase activity

**Figure 5:**
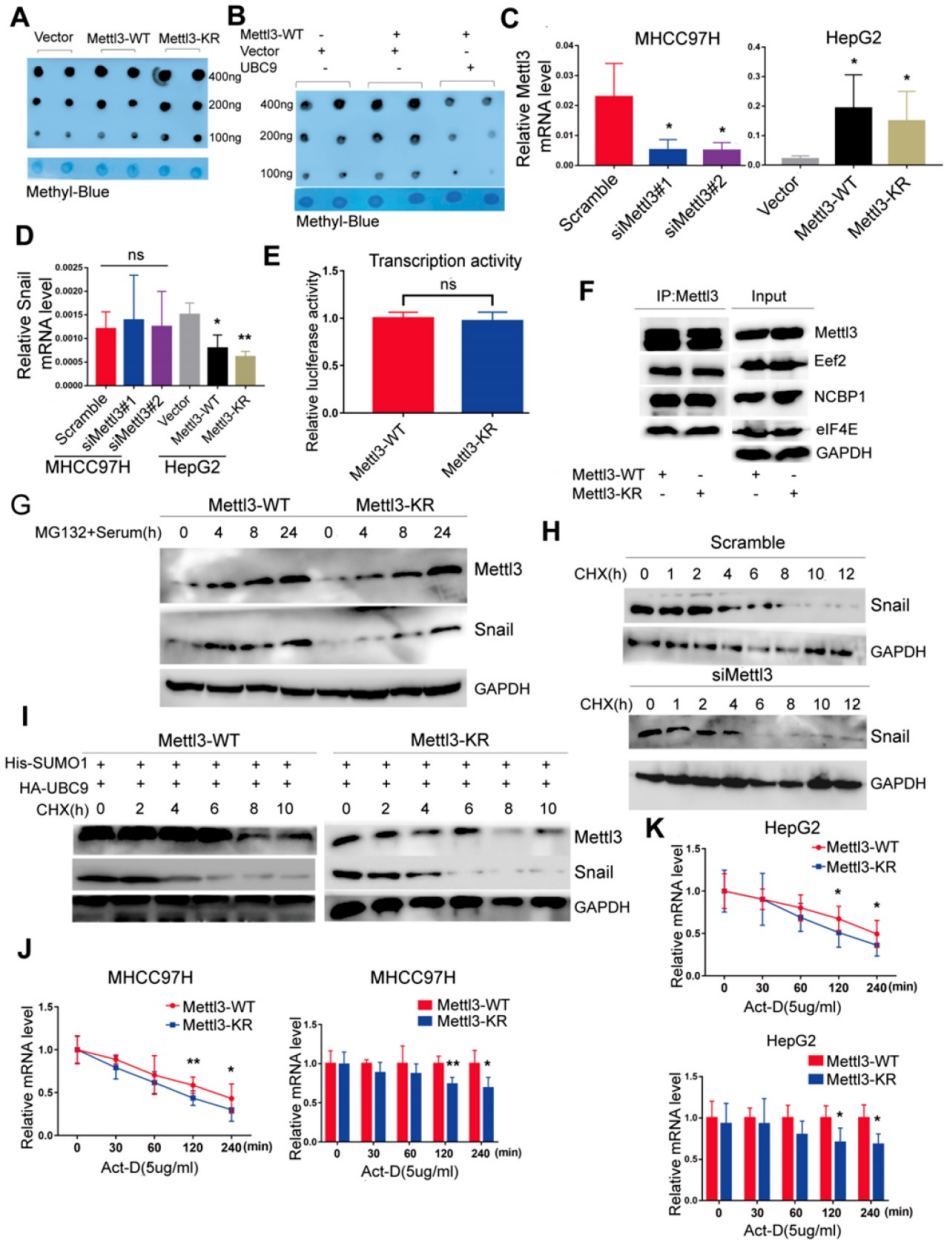
** SUMOylation of Mettl3 regulates Snail mRNA homeostasis via m6A methyltransferase. (A)** Mettl3-WT or -KR was transiently transfected into MHCC97H cells and detected by the dot-blot assay with the anti-m6A antibody. Equal loading of mRNAs was confirmed by methylene blue staining. **(B)** Mettl3 with or without Ubc9 was transfected into MHCC97H cells and detected by the dot-blot assay with the anti-m6A antibody. Equal loading of mRNAs was confirmed by methylene blue staining. **(C)** Suppression or overexpression of Mettl3 in HCC was determined by RT-qPCR, and GAPDH was used as the normalized control. **(D)** mRNA of Snail in Mettl3 suppression or overexpression cells. **(E)** Mettl3-WT or Mettl3-KR-expressing cells were transfected with the pEZX-PL01-Snail promoter reporter plasmid and negative control plasmid for 36 h. Results were expressed as the ratios between F-luc and R-luc activities. **(F)** IP immunoblot analysis was performed in Mettl3-WT- and Mettl3-KR-expressing cells with the anti-Mettl3 antibody, followed by western blotting with Mettl3, anti-Eef2, anti-eIF4E, and anti-NCBP1 antibodies. One-tenth of lysates as the input was immunoblotted with indicated antibodies. **(G)** Mettl3-WT- and Mettl3-KR-expressing cells were pretreated with MG-132 for 6 h and stimulated with serum for indicated times. Subsequently, Mettl3 and Snail protein expression levels were analyzed by western blotting. **(H)** Scramble or siMettl3-expressing cells were fed with CHX for the indicated times, and protein expression of Mettl3 and Snail was analyzed by western blotting. **(I)** Mettl3-WT or Mettl3-KR-expressing cells transfected with different plasmids were treated with CHX for the indicated times, and protein expression of Mettl3 and Snail was detected by western blotting. **(J** and **K)** The decay rate of mRNA and qPCR analysis of Snail at the indicated times after exposure to the transcription inhibitor actinomycin D (5 μg/mL) in MHCC97H **(J)** and HepG2 **(K)** cells. The relative expression level was normalized to β-actin. Data are presented as mean ± s.d. * p < 0.05, ** p < 0.01; Student's t-test.

To dissect the molecular mechanism of the effect of Mettl3 SUMOylation on Snail accumulation, we first assessed whether SUMOylation altered its m6A RNA methyltransferase activity. Liver cancer cells transfected with the empty vector, Mettl3-WT, or Mettl3-KR were extracted for the dot-blot assay. The result showed that the m6A modification level in cells transfected with Mettl3-KR was higher than Mettl3-WT-transfected cells (Figure 5A). Similar results were observed after UBC9 overexpression to increase Mettl3 SUMOylation (Figure 5B). Thus, SUMOylated Mettl3 repressed its m6A RNA methyltransferase activity in this study. Simultaneously, we examined Snail mRNA levels. The efficiencies of Mettl3 knockdown by siRNAs against Mettl3 or overexpression by Mettl3-WT or Mettl3-KR plasmid were verified (Figure 5C). However, there was no difference in Snail mRNA expression in Mettl3 suppression cells, and Snail mRNA expression in Mettl3 overexpression cells was significantly inhibited (Figure 5D). Further, there was no difference in promoter activity of Mettl3-WT cells compared to Mettl3-KR cells, indicating that transcription activity of Snail was not affected by m6A (Figure 5E).

We then hypothesized that Mettl3 SUMOylation may regulate Snail expression by affecting its protein stability, translation efficiency, or mRNA stability since Mettl3 can interact with translation initiation factors such as eIF3B, eIF4E, and NCBP1 to enhance translation 17. As such, Mettl3-WT- and Mettl3-KR-expressing cells were harvested, and their cell lysates were subjected to co-immunoprecipitation with anti-Mettl3 or control IgG antibody, followed by western blotting with indicated antibodies (Figure 5F), The results revealed that SUMOylation of Mettl3 did not influence the translation initiation factors and translation elongation factors, suggesting that Mettl3 SUMOylation did not regulate HCC cell translation efficiency. Furthermore, following administration of MG132, a proteasomal protein degradation inhibitor, at various timepoints, serum stimulation elevated and stably maintained elevated Snail expression, and the defective SUMO mutant of Mettl3 did not appear to have a significant effect on its degradation via the proteasome pathway (Figure 5G).

In addition, we investigated the kinetics of Snail degradation following mitogen stimulation in the presence of protein biosynthesis inhibitor cycloheximide (CHX) and tested the effects of depletion of endogenous Mettl3 on the kinetics. Results showed that half-lives of Snail proteins were similar between NC and siMettl3 cells (Figure 5H). Further, in Mettl3-WT and Mettl3-KR cells transfected with both SUMO1 and Ubc9, cycloheximide-based protein stability assays revealed that Mettl3 SUMOylation was not linked to Snail protein stability (Figure 5I). We next sought to assess the RNA decay rate in Mettl3-WT and Mettl3-KR cells treated with the transcription inhibitor actinomycin D to block new RNA synthesis. Snail mRNA expression was decreased, and the Snail mRNA half-lives were significantly shortened in SUMO-mutant Mettl3 cells (Figure 5J-K). Our data suggests that the Mettl3 SUMOylation deficiency impaired Snail homeostasis to promote its degradation and reduced its expression via an m6A methyltransferase activity mechanism.

### Combination of Mettl3 and Snail expression correlates with unfavorable outcomes of HCC

**Figure 6:**
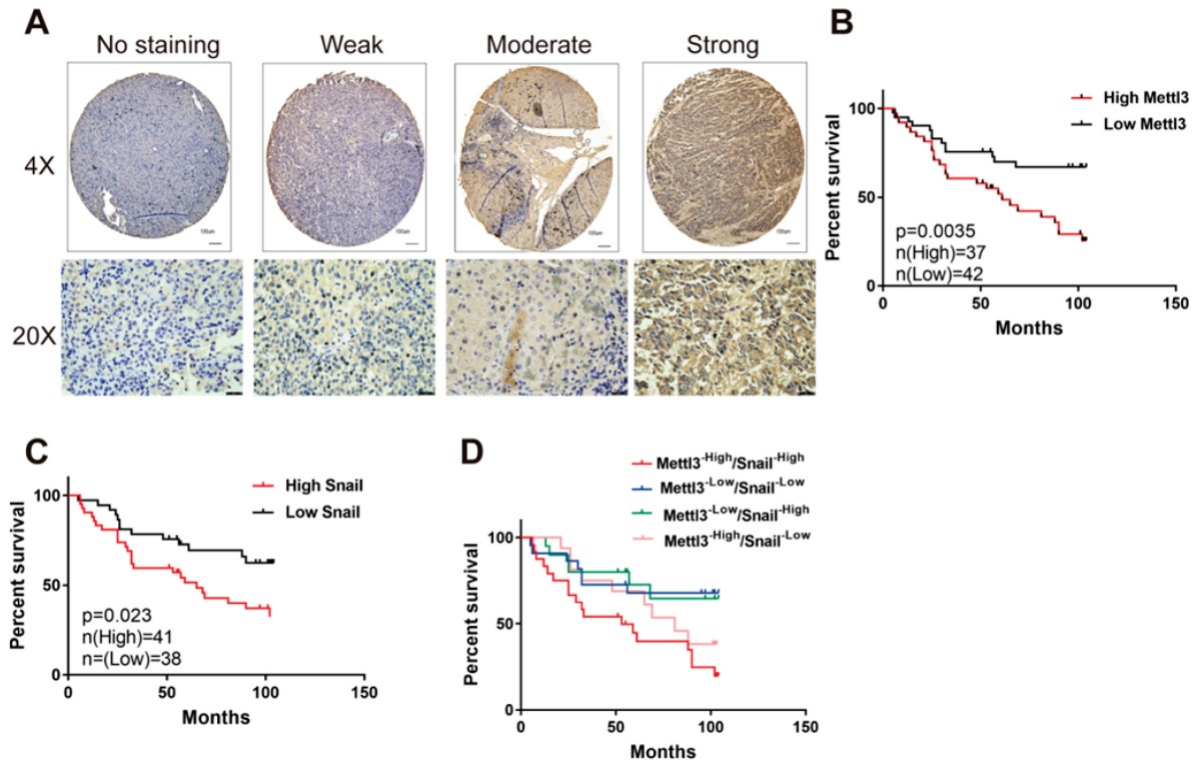
** Elevated Mettl3/Snail level is associated with poor outcomes of HCC. (A)** Mettl3 protein levels in 79 human liver cancer tissues were analyzed by immunohistochemistry. The images show different staining intensities of Mettl3 protein. Scale bars, 100 μm (upper panel), 25 μm (lower panel). **(B-D)** Kaplan-Meier survival curves of overall survival based on Mettl3 **(B)**, Snail **(C)**, or Mettl3 and Snail co-expression **(D)** in HCC patients. Log-rank analysis was employed to compare differences between any two groups.

We first evaluated the prognostic value of Mettl3 expression in HCC samples. As shown in Figure 6A, IHC staining revealed that Mettl3 was localized in the cytoplasm and nucleus of HCC cells. The median value of immunoreactivity scores was used as the cut-off value. In total, 79 HCC patient samples were included and divided into two groups according to Mettl3 expression levels. Multivariate analyses revealed that Mettl3 expression levels, with a hazard ratio (HR) of 3.3405 and a 95% CI of 1.652-7.019, were independent prognostic factors in patients with HCC (Table 1). Kaplan-Meier survival curves and log-rank tests revealed that Mettl3 overexpression levels were significantly associated with overall survival in HCC patients (Figure 6B). The overall survival was longer in patients with lower levels of Mettl3 expression compared to patients with higher levels. Similar results found that high Snail expression predicted poor outcomes of HCC (Figure 6C). Moreover, patients were further divided into four groups: Mettl3^-high^/Snail^-high^, Mettl3^-low^/Snail^-low^, Mettl3^-low^/Snail^-high^, and Mettl3^-high^/Snail^-low^, which further illustrated that HCC individuals with Mettl3^-high^/Snail^-high^ expression tended to have the worst overall survival rates than any other groups (Figure 6D).

## Discussion

SUMOylation as a post-translational modification has been proposed to be an important functional regulatory link to cancer growth and metastasis. SUMOylation proceeds via an enzymatic pathway that is mechanistically analogous to ubiquitination but requires a different E1-activating enzyme and a SUMO-specific E2-conjugating enzyme. The regulatory mechanisms of SUMOylation in cancer progression and metastasis remain unclear.

However, multiple reports have indicated that m6A mRNA modification participates in multiple biological processes and in cancer cell progression 8, 15, 16, 33-36. Through functional interplay among m6A methyltransferases and demethylases, accumulating evidence has indicated that the dynamic m6A modification contributes to the pathogenesis of various diseases, including HCC. Although Mettl3 is the most important component of the RNA m6a methyltransferase complex, few studies have directly focused on the role of Mettl3 SUMOylation modification in human diseases, especially in cancers. Thus, in this study, we aimed to explore the role and mechanism of Mettl3 SUMOylation on HCC progression.

Although Mettl3 has exhibited several functions in cancer cells, some studies have shown conflicting results, such as both high and low expression of m6A promoted tumor progression of acute myeloid leukemia through diverse downstream targets 14, 35. Further, knockdown of Mettl3 resulted in reduced self-renewal abilities in mouse embryonic stem cells 17, whereas Geula et al. reported that Mettl3 facilitated resolution of naive pluripotency towards differentiation 36. Additionally, Mettl3 has been shown to act as a tumor suppressor in renal cell carcinoma 18. Moreover, studies have stated that either high or low expression of Mettl3 could promote tumorigenicity and self-renewal of glioma stem-like cells 37, 38.

These discrepant findings emphasize the complexity of m6A modification and demonstrate that the roles of Mettl3 post-translational modifications in cancer progression and the precise regulatory mechanisms are poorly understood. A study reported that N6-methyladenosine modulated the Snail messenger RNA translation efficiency 29. Further, Mettl3 knockdown resulted in an overall decreased translation efficiency of YTHDF1 target transcripts 39. Du et al. reported Mettl3 modification by small ubiquitin-like modifiers SUMO1 at K177, K211, K212, and K215 both *in vitro* and *in vivo*, which promoted tumorigenesis in H1299 cells 24. Considering the differing results of Mettl3 and m6A in various cancer types, our study may provide the causative link between Mettl3 SUMOylation and tumorigenesis and metastasis.

Further, our study confirmed Mettl3 SUMOylation at multiple sites, which is essential for its oncogenic properties. Increased SUMOylation of Mettl3 promoted HCC cell growth and metastasis, which was repressed in the SUMO-site mutant Mettl3-KR. Moreover, mitogen stimulation triggered Mettl3 SUMOylation and enhanced SUMO1 conjugation of Mettl3 through UBC9 upregulation, and this response showed a positive correlation to liver cancer with high metastatic potential. Additionally, SUMOylation-deficient Mettl3 suppressed Snail accumulation, while restored Snail expression in defective SUMO mutant of Mettl3 in HCC cells attenuated the suppression of proliferation and metastasis *in vivo* and *in vitro*. Although Mettl3 SUMOylation did not alter its protein stability and localization or transcription activity, and translation efficiency of Snail, it regulated Snail mRNA homeostasis in an m6A methyltransferase activity-dependent manner to enhance HCC cell growth and metastasis.

m6A modification processes have been reported to be involved in all stages of the RNA life cycle, including nuclear export, post-translation modulation, and mRNA stability 40-42. m6A was found to promote RNA export from the nucleus to the cytoplasm; however, m6A downregulation delayed mRNA export 43. Further, reports have shown that Mettl3 knockdown promoted WTAP mRNA stability but had no effect on CDCP1 mRNA stability 44, suggesting a negative impact of m6A on mRNA stability. Another study demonstrated that high expression of FTO, m6A demethylases, mediated the repression of ASB2 and RARA mRNA stability 35. In addition, Zhang et al. reported that m6A upregulation by ALKBH5 knockdown did not affect FOXM1 mRNA stability 45. Nevertheless, this study revealed the underlying functions of Mettl3 SUMOylation involved in HCC cell growth and metastasis through regulation of Snail mRNA homeostasis. Considering the discrepant findings of m6A or Mettl3 on mRNA homeostasis in different disease types, we speculate that it might be related to target or spatial location specificity. However, detailed mechanisms underlying m6A methylation and mRNA fate require further study.

In summary, this study proposed a potential regulatory mechanism involving UBC9, SUMOylated Mettl3, and Snail in HCC progression, suggesting Mettl3 SUMOylation as a promising diagnostic and therapeutic target for liver cancer treatment.

## Supplementary Material

Supplementary figures.

## Figures and Tables

**Table 1:**
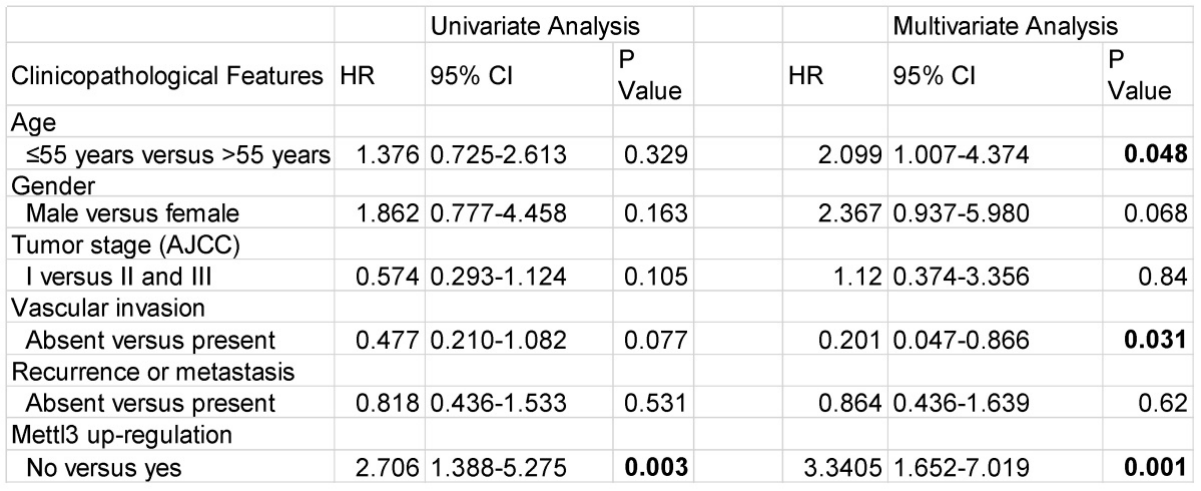
Univariate and multivariate analysis for overall survival in HCC patients

Abbreviations: HR, hazard ratio; CI, confidence interval. P-values were calculated by log-rank test. Bold values indicate statistically significant values.

## Abbreviations

EMT: epithelial-to-mesenchymal transition
HBV: hepatitis B virus
HCV: hepatitis C virus
HGF: hepatocyte growth factor
HR: hazard ratio
TCGA: the cancer genome atlas
HCC: hepatocellular carcinoma
m6A: N6-methyladenosine
Mettl3: Methyltransferase-like 3
EMT: epithelial-to-mesenchymal transition
3 UTRs: 3 untranslated regions
SUMO: small ubiquitin-like modifier
PTM: posttranslational modification
