# Supplementary material for: SUMO1 modification of methyltransferase-like 3 promotes tumor progression via regulating Snail mRNA homeostasis in hepatocellular carcinoma
Source: Theranostics. 2020 Apr 27;10(13):5671–86. doi: 10.7150/thno.42539 (PMC7254988; doi:10.7150/thno.42539)
Supplement: Supplementary file 1 — Supplementary figures. [file thnov10p5671s1.pdf]

SUPPLEMENTARY FIGURE

Figure S1 SUMO1 and Ubc9 with or without Senps were transfected into type Mettl3 or SUMOylation-defective Mettl3-(KR) expressing cells in the presence of serum or not, followed by the IP immunoblot assay for detection of SUMOylated bands with anti-METTL3 antibody.

Figure S2 A and B IP immunoblotting analysis examining SUMOylation of endogenous Mettl3 from whole-cell extracts after incubating in serum-containing or serum-free medium with anti-SUMO1 antibody **(A)** or anti-Mettl3 **(B)** antibody, followed by western blotting with anti-UBC9 and anti-Mettl3 antibody.

Figure S3 IP immunoblot analysis was conducted with the anti-Mettl3 antibody and whole-cell extracts from PLC/PRF/5 or HCCLM3 cells stimulated with or without serum. Cells were harvested and subjected to co-immunoprecipitation with the anti-Mettl3 or control IgG antibody, followed by western blotting for detection of SUMOylated bands with anti-METTL3 antibody.

Figure S4 Effects of Mettl3-WT-, Mettl3-KR-, Snail- and Mettl3-KR/Snail-expressing cells on cell migratory ability were detected by wound scratch assay. The area of wound scratch in response to serum stimulation is shown, with 100% representing the control at 0 h. Data are presented as mean ± s.d. * $p < 0.05$, ** $p < 0.01$; Student's t-test.

Figure S1

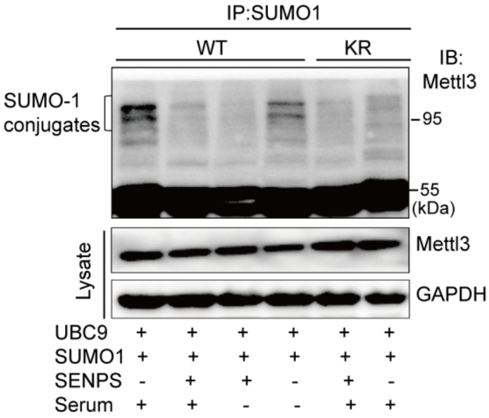

Figure S2

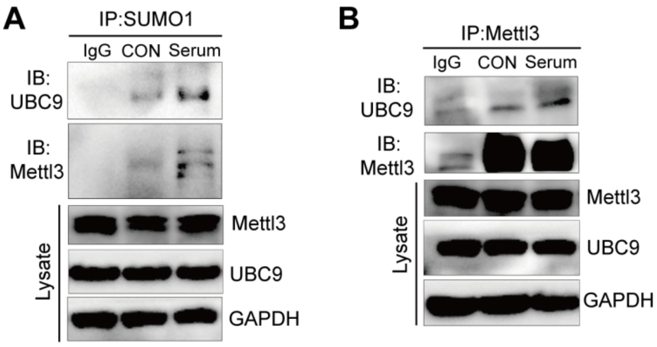

Figure S3

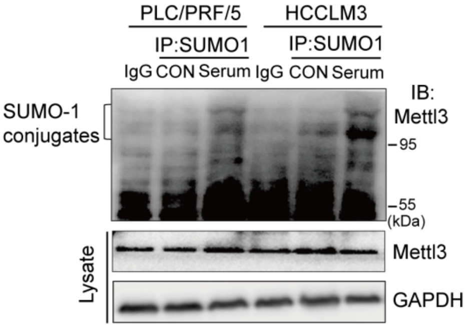

Figure S4

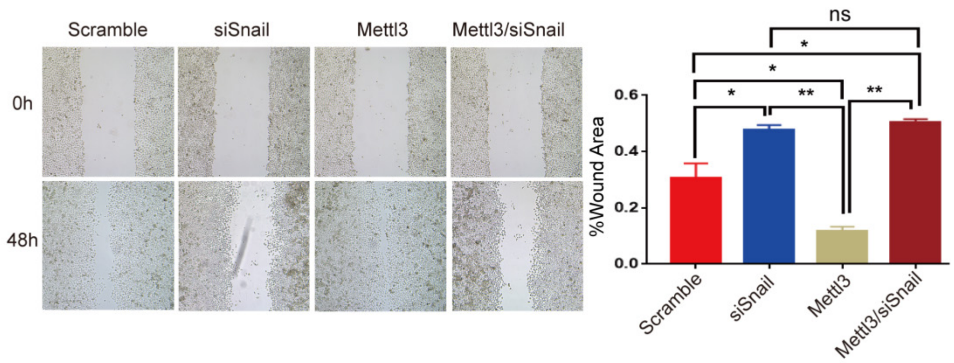

Scramble
siSnail
Mettl3
Mettl3/siSnail
0h
48h
ns
*
*
*
**
**
%Wound Area
0.6
0.4
0.2
0.0
Scramble
siSnail
Mettl3
Mettl3/siSnail
